# Cardiac Autonomic Dysfunction in Multiple Sclerosis: A Systematic Review of Current Knowledge and Impact of Immunotherapies

**DOI:** 10.3390/jcm9020335

**Published:** 2020-01-24

**Authors:** Oliver Findling, Larissa Hauer, Thomas Pezawas, Paulus S. Rommer, Walter Struhal, Johann Sellner

**Affiliations:** 1 Department of Neurology, Kantonsspital Aarau, 5001 Aarau, Switzerland; oliver.findling@ksa.ch; 2 Department of Neurology, University Hospital Tulln, Karl-Landsteiner-University, 3420 Tulln, Austria; walter.struhal@tulln.lknoe.at; 3 Department of Psychiatry, Psychotherapy and Psychosomatic Medicine, Christian Doppler Medical Center, Paracelsus Medical University, 5020 Salzburg, Austria; larissa.sellner@salk.at; 4 Division of Cardiology, Department of Internal Medicine II, Medical University of Vienna, 1090 Vienna, Austria; thomas.pezawas@meduniwien.ac.at; 5 Department of Neurology, Medical University of Vienna, 1090 Vienna, Austria; paulus.rommer@meduniwien.ac.at; 6 Department of Neurology, Landesklinikum Mistelbach-Gänserndorf, 2130 Mistelbach, Austria; 7 Department of Neurology, Klinikum rechts der Isar, Technische Universität München, 81675 München, Germany; 8 Department of Neurology, Christian Doppler Medical Center, Paracelsus Medical University, 5020 Salzburg, Austria

**Keywords:** cardiac autonomic dysfunction, fingolimod, heart rate variability, multiple sclerosis, orthostatic intolerance

## Abstract

Cardiac autonomic dysfunction (CAD) has been reported in patients with multiple sclerosis (MS). This systematic review summarizes the evidence for the types and prevalence of CAD in MS patients, as well as its association with MS type, disease characteristics, fatigue and immunotherapies used to treat MS. The analysis revealed that CAD is correlated with pathophysiological processes of MS, can trigger serious cardiovascular complications that may reduce life expectancy, and may have implications for treatment with immunotherapies, especially fingolimod. Numerous mainly small case–control or cohort studies have reported various measures of CAD (particularly heart rate variation) in MS patients, showing higher rates of abnormality versus controls. A smaller number of studies have reported on cardiac autonomic symptoms in MS, including orthostatic intolerance/dizziness in around 50% of patients. CAD also appears to be associated with disease duration and to be more common in progressive than relapsing–remitting MS. However, although a substantial evidence base suggests that assessing CAD in people with MS may be important, standardised methods to evaluate CAD in these patients have not yet been established. In addition, no studies have yet looked at whether treating CAD can reduce the burden of MS symptoms, disease activity or the rate of progression.

## 1. Introduction

The autonomic nervous system maintains homeostasis of heart rate (HR), blood pressure (BP), respiration, and bowel and bladder control, amongst others. Autonomic dysfunction can lead to a wide range of symptoms, including HR and BP dysregulation, as well as pulmonary symptoms and bowel or bladder dysfunction. Autonomic dysfunction has been reported in patients with multiple sclerosis (MS); however, although bowel and bladder problems are commonly listed as symptoms of MS, cardiac dysfunction in MS is less well understood. Cardiac autonomic dysfunction (CAD) may include orthostatic intolerance and orthostatic hypotension due to baroreflex dysfunction, as well as postural tachycardia syndrome or reduced heart rate variability [1]. CAD in MS is often detectable with detailed autonomic testing, may or may not be symptomatic, and may be associated with the presence of brainstem lesions [2].

In MS, CAD is most likely caused by central dysregulation of sympathetic and parasympathetic outflow to the cardiovascular system. Sympathetic activity may be increased significantly during the early stages of MS, while later disease stages may lead to progressive noradrenergic failure and adrenergic receptor dysfunction [3]. It has been assumed that with increasing impairment of the autonomic nervous system during MS, sympathetic or parasympathetic modulation alters immune responses, causing a vicious neuroinflammatory cycle. However, it is unclear whether those alterations are associated with the structural damage to the CNS in MS or an epiphenomenon of disturbed autonomic feedback cycles. Sympathetic and parasympathetic dysfunction display different patterns over the course of MS [3]. It has been suggested that parasympathetic dysfunction correlates with disease progression and disability, and its impairment could be the consequence of MS. Sympathetic dysfunction, on the other hand, is associated with inflammation and clinical activity of MS and may have a pathogenetic role in the development of MS [3,4,5]. MS activity has also been linked with changes in the expression of different receptors responsible for the communication between immune cells and the sympathetic autonomic system—for example, in the expression of dopaminergic and adrenergic receptors or of different subunits of the nicotinic acetylcholine receptor in the peripheral nervous system [6].

As CAD has been correlated with landmark pathophysiological processes of MS, such as underlying inflammation and neurodegeneration, identifying this type of autonomic dysfunction may be of critical importance [7]. Five non-invasive tests often used to assess CAD are orthostatic BP response, immediate orthostatic HR response (30/15 ratio), heart rate variation (HRV) during deep breathing, Valsalva ratio and BP response to sustained handgrip [8]. Subjective, clinical effects of autonomic dysfunction in MS are assessed using the Composite Autonomic Symptom Score (COMPASS-31). Autonomic function overall can be measured using the Composite Autonomic Scoring Scale (CASS).

With growing evidence of the presence of CAD in patients with MS, the question arises whether CAD contributes to reduced life expectancy and severe cardiovascular complications. Data from a large Danish MS cohort (*n* = 13,963) show a slightly increased risk of acute myocardial infarction, stroke and heart failure within the first year after the first diagnosis of MS compared with the general population; during long-term follow-up, the increased risk of stroke and heart failure persisted [9]. CAD varies by patient but may give rise to baroreflex dysfunction with orthostatic intolerance and orthostatic hypotension, postural tachycardia syndrome or decreased HRV [10]. Patients who experience these symptoms are likely to have an increased risk of cardiovascular complications and may have a lower life expectancy than other patients with MS [10,11]. Furthermore, acute cardiovascular episodes could be the first clinical manifestation of MS, although cardiologists and neurologists may not recognise them as being centrally mediated [12]. This is illustrated by a case of takotsubo (or stress) cardiomyopathy in a young man with no medical history of note, who did not have myocarditis or acute myocardial infarction but was diagnosed with MS and found to have some lesions that are likely to have affected the central autonomic network [13]. Another recently published case study describes a patient who suffered reverse takotsubo cardiomyopathy thought to be linked to an MS relapse [14]. Accordingly, there is an increasing awareness of the need to monitor sympathetic and parasympathetic input to the heart in patients with MS; eventually, this may prevent severe CAD complications [15,16].

In addition to the above considerations, there is some indication that CAD is linked to disease activity in patients with clinically isolated syndrome (CIS). In a prospective study with a 2-year follow-up of autonomic function using the CASS in patients with CIS (*n* = 62), MRI activity was linked to CAD [17]. In another study, data from 94 patients with CIS over 3 years’ follow-up showed that autonomic dysfunction, including CAD, can be an independent predictor of the development of the first relapse after CIS. The authors identified three predictors of relapse: COMPASS-31 >7.32, more than three T2 lesions (both increasing the likelihood of relapse) and a decrease in supine epinephrine concentrations (decrease in the likelihood of relapse) [18]. CAD may also have implications for the treatment of MS as it can be induced or exacerbated by medication used in MS therapy, as exemplified by case reports of life-threatening cardiac complications in MS patients treated with fingolimod, including Mobitz type heart block, sustained bradycardia and one case of sudden cardiac death [19,20,21,22].

The objective of this systematic review is to summarise current knowledge on CAD in MS patients and the effect of immunotherapies for MS on cardiac autonomic function.

## 2. Literature Search

A search of PubMed was conducted in September 2019, using the term “multiple sclerosis” in combination with “cardiac autonomic”, “heart rate variability”, “orthostatic hypotension”, “exercise intolerance”, “bradycardia”, “QT interval”, “arrhythmia” or “syncope”. Articles published in 1990 or earlier were not considered for inclusion. Titles, abstracts and full-text articles were reviewed for inclusion. Reference lists of review articles were examined for further articles for possible inclusion. Case reports, case-control or cohort studies, retrospective data analyses, and open-label or randomised clinical trials reporting information on CAD in MS patients were included.

**Figure 1:**
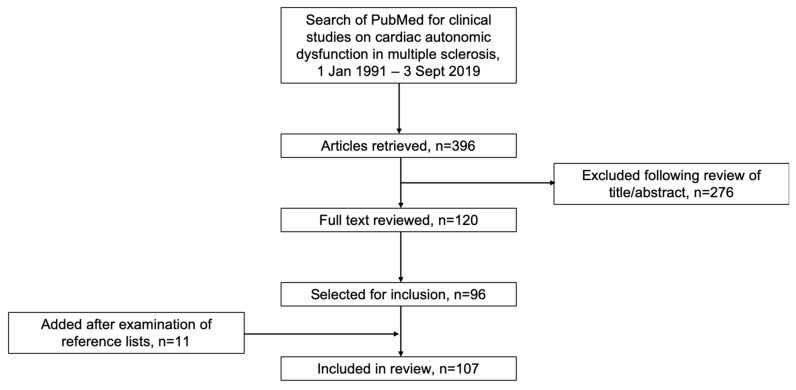
Flowchart of the search strategy.

The search retrieved 396 articles, of which 120 were selected for review of full text. Of these, 88 were included in the review. A further nine articles were added following examination of reference lists of review articles, giving 97 articles for inclusion in the review (Figure 1). Supplementary Materials File S1 provides an overview of the excluded articles. Information extracted from included articles included the types, prevalence and causes of CAD in MS and its association with patient/disease characteristics and immunotherapies used to treat MS.

The article recognises that terms such as heart rate variability and others are a cross-reference to a number of different methods and algorithms performed. Therefore, this review also serves as a reference to the diversity of autonomic test protocols available.

## 3. CAD in MS

### 3.1. Testing for CAD in MS

Several studies have considered the best methods to test for CAD in MS patients, and some have combined methods for testing for CAD with other autonomic test methods such as quantitative sudomotor axon reflex test to make more general statements about the involvement of the autonomic nervous system. Some authors used conventional electrocardiographic (ECG) parameters to make statements about the autonomic dysfunction of the cardiovascular system. Significantly higher maximum P-wave duration and P-wave dispersion (risk factors for atrial fibrillation) were reported in 31 MS patients compared with 33 sex- and age-matched controls [23], and similar findings (along with a non-significant increase in QT interval) were reported from a study in 84 MS patients and 84 age- and sex-matched controls [24]. An Italian study in 31 relapsing–remitting MS (RRMS) patients reported that 16% had an increased QTc interval, which seemed to have a cerebral origin possibly driven by involvement of the insular cortex. The authors concluded that autonomic dysfunction is responsible for changes in ECG [25].

Other authors used Ewing’s Battery, an established combination of methods to diagnose autonomic impairment of the cardiovascular system, or parts of it (Table 1). A study in 33 MS patients and 58 healthy controls used Ewing’s Battery (Valsalva ratio, deep breathing test, 30/15 ratio, BP variation after change of posture, handgrip test) and QTc. They found an abnormal 30/15 ratio and an abnormal Valsalva response. However, they noted that the presence of an abnormal 30/15 ratio and an abnormal Valsalva response does not necessarily imply clinically significant cardiovascular dysfunction. They concluded that autonomic dysfunction may be subclinical and that it is preferable to combine several tests for a more thorough and accurate evaluation [26].

An advanced and complex analysis technique for the investigation of CAD is the frequency domain method or power spectrum analysis because it gives extended information—for example, on the evaluation of 24-h ECGs [35,37,38,49]. Two studies in the 1990s suggested that spectral analysis of HR and/or arterial BP oscillations may considerably improve the pathophysiological interpretation of CAD in MS patients [30,31]. A study from 2004 reported similar findings for spectral analysis of HRV in rest-tilt-rest tests [32]. A 1995 study suggested that tests of HRV performed during sleep may be more sensitive to detect reduced parasympathetic activity than standard tests during wakefulness for detecting CAD in MS patients [33]. A more recent study in 20 RRMS patients and 20 age- and sex-matched controls compared the performance of the classical standardised Ewing battery with newer methods based on heart rate variability and spontaneous baroreflex function in both time and frequency domain. In addition, the authors performed a short-term analysis of HRV by recording only the last 10 min of resting ECG recording. They concluded that short-term analysis of HRV may be more useful than the standard Ewing battery of tests, showing both parasympathetic and sympathetic impairment in MS patients (*p* < 0.05) [28]. An earlier study in 18 MS patients and 20 healthy controls, also using the frequency domain technique, suggested that vagal power, the respiratory component of HRV, could be an alternative non-invasive measure [29].

One study used the Lyapunov exponent, which is a measure of a system’s predictability and sensitivity to changes in its initial conditions, to assess heart rate dynamics [38]. The authors found lower Lyapunov exponents in MS patients (*n* = 11) than in healthy controls (*n* = 11), suggesting that MS patients display a more stable and thus less adaptive central autonomic organisation [38].

Since the availability of sophisticated tests such as spectral analysis after Valsalva or tilt-table test is limited, another study tried to find evidence of CAD by monitoring HR during a cycle test. Examining 31 MS patients and 31 age-matched controls revealed that the pattern of increase in HR on starting a simple cycle test was different in some participants with MS who had been shown to have autonomic dysfunction on laboratory testing, highlighting that HR responses to dynamic exercises may be compromised in some people with MS. The authors suggested that physiotherapists should monitor the HR response to a dynamic exercise test in people with MS before prescribing an exercise programme to ensure patients’ safety [27].

In a number of studies, the above-mentioned techniques had been combined.

### 3.2. Prevalence, Findings and Types of CAD in MS

A meta-analysis published in 2015 (16 studies, 611 MS patients) reported a prevalence of CAD of either 42.1% or 18.8% depending on whether only one or at least two abnormal autonomic test results were used to define autonomic dysfunction [7].

Impaired HRV, measured in the time or the frequency domains, is one of the most commonly reported findings indicating CAD in MS patients. Studies in the 1990s found lower HRV (including HRV related to body movements during sleep) in MS patients, caused by a significant increase in sympathetic cardiovascular tone [35,36]. Later, significant reductions in various long-term (24-h Holter monitoring) HRV measures were reported in 34 MS patients compared with 20 healthy controls (*p* < 0.05) [37].

A study in the early 1990s took a different approach, finding that the HR dynamics of young, stable MS patients (*n* = 11) were characterised by significantly lower Lyapunov exponents than healthy controls (*n* = 11), suggesting that MS patients, from an early and even relapse-free stage of illness, display a more stable and thus less adaptive central autonomic organisation [38].

In a Finnish study (51 MS patients, 50 controls), HR responses to the deep breathing test (*p* < 0.05) and the tilt-table test (30:15 ratio; *p* < 0.001) were significantly lower in MS patients than in controls [43]. An Australian study investigating spontaneous changes in BP and HR showed diminished baroreceptor sensitivity in 23 MS patients compared with 23 age- and sex-matched controls (*p* < 0.05), with the authors suggesting that this provides valuable information on the interaction of sympathetic and parasympathetic activity in assessing the overall autonomic function [4]. Measurement of cardiac autonomic function during postural changes and exercise in 23 MS patients and 20 age-matched controls showed that parasympathetic indexes were significantly lower (*p* < 0.05) in MS patients during baseline sitting and post-exercise recovery, whereas sympathovagal parameters were similar in both groups [44].

Impaired heart ejection fraction as a finding possibly related to impairment of cardiovascular autonomic function was reported by Olindo et al. They used radionuclide angiocardiography to assess right and left ventricular ejection fractions (VEFs) at rest, observing a significant decrease in right (*p* = 0.025) and left VEF (*p* < 0.0001) in 40 MS patients compared with 40 controls; pathological results were recorded for right (7.5%), left (10%) and both (7.5%) VEFs in 25% of MS patients [75].

### 3.3. Symptoms of CAD in MS

Even in the presence of pathological findings such as altered HRV, CAD can be subclinical [27,42]. However, orthostatic symptoms, which are among the symptoms commonly associated with CAD, were reported in 61% of 104 patients with CIS in a study from Croatia, and in 24% of 100 MS patients in a Turkish study [39,76]. Nevertheless, in both studies, the authors did not distinguish between different forms of orthostatic dysfunction such as dizziness, palpitation or syncope. This was, however, done in an Iraqi study: in 55 MS patients and 40 age-matched controls, postural dizziness was present in 67% of patients and 33% of controls, syncope/pre-syncope in 18% and 0%, and palpitations in 64% and 23%, respectively [40]. Another Turkish study in 22 RRMS patients and 22 age-matched controls reported orthostatic dizziness in 9 (54%) patients and 6 (27%) controls [41]. In a German study (*n* = 40; 27 RRMS, 13 secondary progressive MS [SPMS]), 50% of the patients reported orthostatic dizziness compared with 17% of controls (*p* < 0.006) [77].

Additionally, a US study looked at nine patients with postural orthostatic tachycardia syndrome (POTS) and MS; all also had fatigue and palpitations, as well as orthostatic dizziness, and five had syncope. Two of the patients had developed POTS 3.5 and 1.5 years before the onset of MS, while the other seven developed POTS over a mean period of 22 months from diagnosis [78].

As a possible clinical complication of CAD, rare cases of Brugada pattern ECGs and takotsubo (or stress) cardiomyopathy have been reported [13,14,15,16,79].

There have been occasional case reports of cardiac symptoms associated with MS relapses. A case was reported of a 20-year-old woman who developed sinus bradycardia (HR 40 bpm) during an MS relapse; no cardiac pathology was found and symptoms gradually improved after a 5-day course of corticosteroid therapy [80].

### 3.4. Association with MS Type and Disease Characteristics

Several studies have looked at CAD in relation to type of MS, disease duration and other characteristics. CASS score and HRV were assessed in 40 patients with RRMS and 30 with progressive MS, showing that those with progressive MS had significantly lower total CASS scores (*p* < 0.001) [45]. Both disease duration and Expanded Disability Status Scale (EDSS) score were positively correlated with total CASS score (both *p* < 0.001), and type of MS, corrected for age, sex and disease duration, was a significant predictor of CASS score (*p* = 0.019). Moreover, HRV was overall lower in patients with progressive MS than in those with RRMS, with a blunted sympathetic reactivity [45]. A similar finding was reported from a study in 84 patients with RRMS, 36 with progressive MS and 60 age- and sex-matched controls [81]. Progressive patients showed altered HRV compared with both healthy controls and RRMS patients. The authors concluded that autonomic balance appears to be intimately linked with both the inflammatory activity of MS, represented by overall hypoactivity of the sympathetic nervous system and its compensatory plastic processes, which appear to be inefficient in cases of worsening and progressive MS [81]. Another study also reported more orthostatic symptoms, orthostatic hypotension and ECG RR-interval variation abnormalities in patients with progressive MS (*n* = 50) than RRMS (*n* = 25) and healthy controls (*n* = 15) [46].

A Turkish study using five non-invasive tests of CAD found abnormal results in 10/22 RRMS patients and 0/22 age-matched controls [41]. Disease duration was significantly correlated with autonomic impairment (*p* = 0.0001) in this study, but EDSS was not, although another Turkish study (*n* = 100) found significantly higher rates of self-reported orthostatic symptoms in patients with higher EDSS scores (3% for EDSS <4 vs. 67% for EDSS >4; *p* < 0.0001) [76]. A Finnish study also found positive correlations between EDSS and several CAD tests [43]. An earlier study evaluated myocardial muscle function, finding significant decreases in ejection fraction and cardiac output in the supine position in 12 MS patients with EDSS 5–7 compared with 12 with EDSS 3–4 and 12 healthy controls, with intensified symptoms in the erect position accompanied by decreased values of stroke volume. The authors suggested that these findings may partly be accounted for by secondary myocardial injury in the course of MS [82].

Impairment of autonomic control of HR was also found to be associated with disease duration (≤5 vs. >5 years: *p* ≤ 0.01 for all HRV variables except low- to high-frequency ratio) in a study including 39 patients with RRMS [49]. However, another study showed that HRV was already decreased in 51 patients with newly diagnosed RRMS compared with 44 age- and sex-matched controls [50]. On the other hand, a slightly diminished sympathetic response to a mental stress test, but unchanged response to orthostasis, was seen in 19 newly diagnosed untreated MS patients compared with 19 age-, sex- and body mass index-matched controls [51]. In the 1990s, one study in 46 MS patients (20 RRMS, 26 SPMS) found that orthostatic HR response showed significant worsening over 1 year [52], while another reported worsening of HRV on deep breathing and immediate orthostatic HR response over 2 years in 20 RRMS patients [53]. The authors suggested that CAD tests may be useful as a surrogate outcome measure for demonstrating subclinical disease progression in MS, as they found over a 2-year period a significant deterioration in CAD tests, but no significant deterioration in EDSS and no significant increase in T2 lesion load on MRI.

A study of 26 patients with clinically active RRMS, 9 with clinically stable RRMS and 24 healthy controls assessed parasympathetic and sympathetic CAD [5]. The number of patients with at least one abnormal sympathetic test was higher in the “active” patient group (39%) than in healthy controls (8%; *p* < 0.02) or “stable” patients (0%; *p* < 0.04), while no difference was seen in the parasympathetic score. Standard autonomic tests were repeated at 3, 6, 12, 18 and 24 months in 18 of the active patients; parasympathetic but not sympathetic dysfunction increased slightly during the follow-up period, with a significant correlation with the increase in clinical disability (r = 0.7; *p* < 0.002). During acute exacerbations, only parasympathetic dysfunction tended to increase in parallel with a deterioration in the EDSS [5].

Two retrospective studies explored cardiac repolarisation, assessed as HR corrected QT intervals, in relation to aspects of MS. In one study of 58 RRMS patients, cardiac repolarisation was prolonged and HR increased during the disease course in patients with motor but not with sensory onset symptoms [83]. The other study, in 43 RRMS patients, found that deterioration of cardiac autonomic regulation during the disease course was associated with disabling but not with benign RRMS [84].

### 3.5. Association with Fatigue and Cognitive Impairment

A study in 84 MS patients used five established autonomic tests and three different fatigue questionnaires to investigate the relationship between CAD and fatigue [8]. Moderate cardiovascular disturbances were found in 17% of the patients and severe cardiovascular autonomic abnormalities in 11%; 64% of the patients were categorised as being fatigued. In 19% of patients, signs of autonomic failure and fatigue were co-existent. Only weakly significant correlations were found between some single autonomic test parameters and fatigue scores, which were confounded by age effects. The authors concluded that autonomic disturbances might contribute to fatigue symptoms in a subgroup of MS patients, but the overall influence of CAD on fatigue seems to be of minor relevance [8]. In a more recent study of 70 patients with early-stage MS, results of the Modified Fatigue Impact Scale (MFIS) correlated with those of COMPASS-31 (r_s_ = 0.607; *p* < 0.001). Multiple regression analysis showed that COMPASS-31 was an independent predictor of fatigue, indicating a link between fatigue and subjective perception of autonomic function; however, only weak correlations were shown between objective assessments of autonomic function and MFIS scores [54].

A study in 50 MS patients (28 with fatigue) using five non-invasive cardiovascular tests reported a loss in ability to increase BP with sustained handgrip in patients with fatigue, suggesting a sympathetic dysfunction (increase of 14.62 ± 9.13 mmHg for the group with fatigue versus 21.68 ± 7.18 mmHg for the non-fatigue group; *p* < 0.05) [55]. In another study, MS patients with fatigue (*n* = 27) had reduced orthostatic HR response (30/15 ratio) and handgrip BP response compared with age- and sex-matched controls (*n* = 36; *p* < 0.0001) and MS patients without fatigue (*n* = 33; *p* < 0.025), while no difference was seen between the latter two groups [56]. The authors concluded that these results may be due to a sympathetic vasomotor lesion with intact vagal heart control [56]. On the other hand, a study in 10 MS patients and 10 age-matched controls concluded that patients with MS who have chronic fatigue show normal sympathetic activity and possibly slightly reduced vagal activity compared with controls. A significant (*p* < 0.05) age-dependent decrease in vagal activity was seen in patients with MS compared with controls [57].

A German study examined responses to cognitive stress in 23 MS patients with fatigue and 25 age- and sex-matched controls [58]. MS patients showed a significantly lower HR increase after the task (*p* < 0.001), and there was a significant negative correlation of the MFIS total score with HR (r = −0.39; *p* = 0.01). In addition, cognitive impairment was associated with a decreased HR reactivity (*p* = 0.02) [58]. A recent investigation of behavioural performance (response bias) and autonomic functioning (HRV and skin conductance level) during an acoustic vigilance task in 53 MS patients suggested that cognitive fatigue in MS is related to parasympathetic activity and reduced responsiveness [59]. Finally, a UK study in 26 MS patients (17 with fatigue) and nine age- and sex-matched controls found that patients with fatigue had reduced levels of alertness and cardiovascular sympathetic activation compared with patients without fatigue and healthy controls. Modafinil showed alerting and sympathomimetic effects in all three groups, which may be how it exerts its anti-fatigue effect in MS [60].

### 3.6. Treatment of CAD in MS

Given the associations described above, improving cardiovascular autonomic nervous system functional activities has the potential to reduce the burden of MS and the rate of complications related to the disease in MS patients with CAD. There are almost no data regarding a specific treatment for CAD in MS patients. In the above-mentioned UK study including 26 MS patients (17 with fatigue), modafinil showed alerting and sympathomimetic effects, but these effects also occurred in non-fatigue patients (*n* = 9) and healthy controls (*n* = 9) [60].

A pilot study in 21 MS patients with symptoms of CAD evaluated a non-drug treatment approach. Indicators of CAD improved 24 h after transvascular autonomic modulation using a combination of balloon angioplasty of anatomically normal veins coupled with external compression during dilation of these veins [61]. Independent studies are needed to confirm the efficacy and safety of this approach, which thus cannot currently be recommended outside of clinical trials.

### 3.7. Possible Mechanisms of CAD in MS

At present, only hypotheses exist regarding the origin of CAD in MS. Compromised carotid baroreceptor control of BP in MS patients may contribute to orthostasis-related symptoms. A study in 10 MS patients showed a diminished ability to increase BP in response to a hypotensive stimulus (neck pressure) compared with 10 sex-, age- and body-weight-matched controls, which was attributed to a blunted vascular conductance response [62]. A study in 13 MS patients and 18 controls evaluated the HR and blood vessel responses to baroreflex stimulation by sinusoidal neck stimulation at different frequencies, showing impaired responses in MS patients [63]. The authors concluded that baroreflex dysfunction is not only restricted to the cardiovagal limb of the baroreflex but that the sympathetic modulation of the blood vessels must also be affected [63].

Recently, a US study in 11 MS patients and six controls reported that intra-neural measurements of muscle sympathetic nerve activity (MSNA) showing reduced MSNA in MS patients may be augmenting impaired autonomic control of cardiovascular function [34].

### 3.8. Association with MRI Lesions

#### 3.8.1. Brainstem/Infratentorial Lesions

It is reasonable to investigate a possible relationship between the presence and localisation of MRI lesions and CAD. Brainstem involvement was reported in a case of orthostatic hypotension causing recurrent syncope in an MS patient in the 1990s. The symptoms were associated with MRI lesions in the paramedian tegmentum and base of the medulla, suggesting the involvement of the nucleus tractus solitarii and bulbospinal sympathetic centripetal pathway, which are closely concerned with cardiovascular autonomic function [85]. Another 1990s case of paroxysmal atrial fibrillation and ECG changes associated with an MS relapse was attributed to a large demyelinating lesion involving the left middle and inferior cerebellar peduncles with extension rostrally into the brainstem, and an active cerebellar lesion was linked to acute bradycardia in an MS patient [80,86].

Two studies reported significant association between presence of CAD and clinical (*p* < 0.02) and MRI (*p* < 0.005) evidence of brainstem lesions while a Finnish study in 51 MS patients and 50 controls reported a significant association (*p* < 0.05) between the total volume of midbrain MRI lesions and impaired BP response [43,47,48]. In contrast, an earlier study from the Netherlands was not able to find a correlation between the number and size of brainstem lesions and abnormalities in autonomic parameters [53].

#### 3.8.2. Supratentorial Lesions

A recent study in 74 MS patients reported associations between a shift of cardiovascular sympathetic–parasympathetic balance towards increased sympathetic modulation and left insular and hippocampal lesions, areas of the central autonomic network [64].

#### 3.8.3. Spinal Cord Lesions

In a French study, both clinical and laboratory-confirmed autonomic dysfunction were associated with spinal cord atrophy on MRI (*p* ≤ 0.03), suggesting that CAD may be more closely related to axonal loss than to demyelinating lesions [46].

Furthermore, in a prospective study of patients with CIS, a 2-year follow-up of autonomic function using CASS (*n* = 62) MRI activity was linked to CAD [17]. However, no significant relationship between location of MRI lesions and HRV analysis was seen in a Turkish study [50].

## 4. CAD Associated with Immunotherapies in MS

### 4.1. Sphingosine 1-Phosphate (S1P) Receptor Modulators

#### 4.1.1. Fingolimod

In a Phase 2 proof-of-concept study of fingolimod (*n* = 281) in MS, HR was reduced by a mean of 13.8 and 16.6 bpm within 6 h after the first dose of fingolimod in patients receiving 1.25 and 5.0 mg, respectively, returning towards baseline with continued treatment [87]. Symptomatic bradycardia within 24 h after the first dose was reported in one patient who received 1.25 mg and three who received 5 mg; all resolved spontaneously. An initial reduction in mean BP (5–6 mmHg lower than the baseline value) within 4–5 h after administration of fingolimod was followed by a sustained elevation (4–6 mmHg higher than baseline) after two months of treatment, with no further increase [87].

Two Phase 3 studies of fingolimod versus placebo (FREEDOMS, *n* = 1272) and interferon beta-1a (TRANSFORMS, *n* = 1292) reported bradycardia and atrioventricular (AV) conduction block at the time of fingolimod initiation [88,89]. Table 2 shows the cardiac findings in the two trials. The HR decreases were maximal at 4–5 h, attenuating after 6 h. Most cases of bradycardia occurred during the first-dose monitoring period, and all resolved within 24 h. In a long-term extension of FREEDOMS (*n* = 920), a transient decrease in HR and a delay in AV conduction were observed in patients who switched from placebo to fingolimod [90]. Symptomatic first-dose bradycardia was seen in two patients, and a transient episode of second-degree AV block on day 1 of therapy was reported in one patient who was asymptomatic and completed the extension study [90].

A further Phase 3 study (FREEDOMS II, *n* = 1083) of fingolimod versus placebo included 24-h Holter ECG monitoring after the first dose and at 3 months [91]. The maximum decrease in mean sitting HR from the pre-dose values was 8.5 bpm in the fingolimod 0.5 mg group and 12.4 bpm in the 1.25 mg group at 5 h after the first dose. The monitoring results are shown in Table 3. Patients receiving 1.25 mg fingolimod in this study were switched to 0.5 mg following review of the data from FREEDOMS and TRANSFORMS [91].

An integrated safety analysis of Phase 2 and 3 studies of fingolimod reported symptomatic bradycardia events in the first 6 h in 9/1640 (0.5%) and 29/1642 (1.8%) patients who received first-dose fingolimod 0.5 and 1.25 mg, respectively. Second-degree AV blocks, usually Mobitz type I (Wenckebach) were observed in 2/1640 (0.1%) patients receiving fingolimod 0.5 mg [92]. Another analysis looked specifically at first-dose effects in the three Phase 3 studies [93]. At nadir, the mean decreases from baseline in the mean sitting HR were 8.1 bpm in the fingolimod 0.5 mg group (*n* = 1212) and 11.3 bpm in the 1.25 mg (*n* = 1219) group. AV node conduction abnormalities were seen in 86 (7.2%) and 153 (12.7%) patients in the 0.5 and 1.25 mg fingolimod groups, compared with 33/773 (4.3%) with placebo and 19/431 (4.5%) with interferon beta-1a. Symptomatic bradycardia on initiation of treatment was reported in 0.6% of patients in the fingolimod 0.5 mg group and 2.1% in the 1.25 mg group. A small decrease in BP was observed in fingolimod-treated patients on day 1 that was maximal 4–5 h after the first dose [93].

A real-world, open-label, single-arm, Phase 3b study (FIRST, *n* = 2417) focused on cardiac safety during initiation of fingolimod 0.5 mg [94]. Bradycardia adverse events occurred in 0.6% of patients and were more frequent in those receiving beta blockers or calcium-channel blockers (3.3%) than in other patient subgroups (0.5%–1.4%); most events were asymptomatic, and all patients recovered without pharmacological intervention. In the 6 h post-dose, the incidences of Mobitz type I second-degree AV block and 2:1 AV block were higher in patients with pre-existing cardiac conditions (4.1% and 2.0%, respectively) than in those without (0.9% and 0.3%). All recorded conduction abnormalities were asymptomatic [94]. In another real-world, open-label, single-arm study (*n* = 906), cardiovascular adverse events occurred in 18 patients in the 6 h following the first dose of fingolimod and included bradycardia (1.3%), first- and second-degree AV block (0.1% and 0.2%), palpitations (0.1%), sinus arrhythmia (0.1%) and ventricular premature beats (0.1%). All events were self-limiting and did not require any intervention. Extended monitoring was required in 34 patients [95].

An ongoing open-label Phase 4 study (START) is using Holter ECG monitoring to assess the safety of the first dose of fingolimod in a large patient cohort [96]. An interim analysis (*n* = 3951) showed that bradycardia and AV conduction abnormalities were rare, transient and benign. Thirty-one (0.8%) patients developed bradycardia (<45 bpm) and 62 (1.6%) patients had second-degree Mobitz I and/or 2:1 AV blocks with the lowest reading of 35 bpm and the longest pause lasting for 2.6 s. No Mobitz II or third-degree AV blocks were observed. After 1 week, there was no second-/third-degree AV block. The researchers concluded that continuous Holter ECG monitoring does not add clinically relevant value to patients’ safety [96].

One small study (*n* = 27) looked at cardiac regulation using 24-h ECG recording after the first dose and again after 3 months of continuous dosing [65]. On the first day, a prolongation of the RR interval (*p* < 0.001), an increase in the values of various HRV parameters (*p* < 0.05 to *p* < 0.001) and a decrease in the low frequency/high frequency ratio (*p* < 0.05) were seen. At 3 months, although the RR interval remained longer (*p* < 0.01), the values of HRV parameters were lower (*p* < 0.01 to *p* < 0.001) than at baseline [65]. Another small study (*n* = 33) looked at HRV shortly before and at 4.5 h and 3 months after the first intake of fingolimod, finding that an initial increase in HRV at 4.5 h was followed by a substantial decrease over 3 months of treatment [66]. A third study (*n* = 64) assessed patients with Holter ECG monitoring and HRV analysis 24 h before, 6 h during and 72 h after fingolimod initiation, as well as at follow-up ≥3 months later [67]. Five (7.8%) patients had new-onset AV block requiring cessation of treatment. HR decreased significantly (*p* < 0.001) on fingolimod treatment, with a nadir at 5 h after initiation, remaining decreased for 72 h; mean HR was still significantly reduced compared with baseline after a mean follow-up time of 14.1 ± 9.6 months (85.0 vs. 75.3 bpm; *p* = 0.002) [67].

Two retrospective database studies have examined the first-dose safety of fingolimod in clinical practice. A US study (*n* = 317) reported that adverse events during first-dose observation were self-limited and included symptomatic bradycardia (*n* = 3), chest tightness (*n* = 2) and hypertension (*n* = 1) [97]. In a Brazilian study (*n* = 180), five patients had high BP and four had right or left branch block before medication; they were kept under observation for longer than 6 h and did not develop complications [98]. Two patients had sinus tachycardia and two had sinus bradycardia at the first ECG registration; they did not develop complications. Twelve (6.7%) other patients were kept under medical observation for longer than 6 h due to symptomatic bradycardia; 3 (1.7%) of them needed intensive care unit treatment for right branch block or second-degree AV block [98]. Another retrospective study (*n* = 212) considered the long-term cardiac safety of fingolimod. In the first 6 h after initiation, 54 (26%) patients had an abnormal ECG and one case of symptomatic second-degree AV block occurred. Over a mean follow-up of 1.5 years, one patient had atrial fibrillation requiring treatment and five had a persistent increase in BP [99].

In addition to the above studies, several case reports have described cardiac complications in MS patients receiving fingolimod. One described a 37-year-old patient with a non-specific complaint of dizziness and asthenia who had a documented episode of ventricular tachycardia on Holter monitoring within the first month of fingolimod treatment [100]. Another case report described a 51-year-old patient who developed a Mobitz type I second-degree AV block during the initial 6-h monitoring period; she then went into a junctional tachycardia and later went back into a symptomatic Mobitz type I AV block with an HR nadir of 38 bpm that was treated with atropine [19]. Another patient had a transient asystole lasting for 7.5 s and sustained bradycardia 21 h after the first dose of fingolimod [20]. Also, a case of sudden cardiac death in the fifth month of fingolimod treatment was reported; postmortem examination showed an extensive contraction band necrosis in the heart, suggesting pre-mortem hypoperfusion due to ventricular arrhythmia. The exact cause of death and relation to fingolimod therapy remains unclear [21].

Other studies have looked at predictors of fingolimod-induced bradycardia. One study (*n* = 55) found significant correlations between measures of parasympathetic function and fingolimod-induced bradycardia; patients with higher Valsalva ratio and HRV during deep breathing had nadir HR ≤ 50 bpm after the first fingolimod dose. Conversely, significant negative correlations were found between measures of sympathetic function and fingolimod-induced PR-interval increase [68]. A sub-study (*n* = 78) of the START study found that low baseline HR was a powerful predictor of fingolimod-induced bradycardia, while another study in 114 patients reported that body mass index, optic nerve involvement, baseline HR and T-peak–T-end interval were independent predictors of greater HR reduction on initiation of fingolimod [69,101]. In a study in 625 patients initiating fingolimod, normalised spectral power in the high-frequency band (marking vagal modulation) and previous annualised relapse rate were independently correlated with the probability of requiring extended monitoring (45 [7.2%] patients) [70]. Finally, in a study in 21 MS patients and 20 normal volunteers, the results of autonomic testing before fingolimod treatment suggested that seven patients who had a delayed HR re-increase after fingolimod initiation had subtle and clinically not overt central autonomic dysfunction [71].

#### 4.1.2. Siponimod

The second S1P receptor modulator approved for treatment of MS was siponimod. Siponimod is selective for S1P receptor subtypes 1 and 5 (compared with fingolimod’s affinity for 1, 3, 4 and 5), and unlike fingolimod, it does not require phosphorylation to function. In a Phase 2 dose-ranging study (BOLD, *n* = 297) [102], patients were randomised 1:1:1:1 to siponimod 10, 2 or 0.5 mg or placebo for 6 months, or 4:4:1 to siponimod 1.25 or 0.25 mg or placebo for 3 months. A dose-dependent decrease in HR on treatment initiation was observed in those who did not receive dose titration (0.5, 2 and 10 mg) [96]. In an extension of the BOLD study (*n* = 252), all patients re-randomised from placebo to the five siponimod doses received dose titration [103]. Only minor decreases in HR from pre-dose levels were observed on starting siponimod and two patients had transient second-degree AV block on 24-h Holter ECG during the dose titration period; no symptomatic bradycardic events were reported during the extension, and there were no clinically significant changes in BP from extension baseline [103].

Initial dose titration (from 0.25 to 2 mg daily over 6 days) also mitigated cardiac first-dose effects in a Phase 3 study (EXPAND, *n* = 1651) of siponimod 2 mg versus placebo [104]. During treatment initiation, bradycardia was reported in 48/1099 (4%) patients receiving siponimod and 14/546 (3%) receiving placebo; bradyarrhythmia (including conduction defects) was reported in 29 (3%) and 2 (0.4%), respectively, and sinus bradycardia in 14 (1%) and 1 (<1%). The maximum reduction in mean HR on day 1 was 5.3 bpm in the siponimod group (4 h post-dose) and 1.2 bpm in the placebo group (1 h post-dose); on day 7, mean reductions were 3.1 and 2.0 bpm, respectively (3 h post-dose) [104].

#### 4.1.3. Ozanimod

A third S1P receptor modulator, ozanimod, binds selectively with high affinity to receptor subtypes 1 and 5 and is undergoing regulatory assessment for the treatment of MS. A Phase 1 cardiac safety study in 145 healthy adults showed that although ozanimod blunted the observed diurnal increase in HR, excursions below pre-dose HR were no greater than with placebo. Ozanimod did not prolong the QTc interval or cause clinically significant bradycardia, suggesting a favourable cardiac safety profile [72]. In the Phase 2 RADIANCE study (*n* = 258), the maximum reduction in mean HR by Holter monitoring during the first 6 h in ozanimod-treated participants was <2 bpm compared with baseline, with no patient having a minimum hourly HR < 45 bpm. ECGs and 24-h Holter monitoring showed no increased incidence of AV block or sinus pause with ozanimod [105]. In the recently reported Phase 3 SUNBEAM trial (*n* = 1346), no first-dose, clinically significant bradycardia or second-degree or third-degree AV block was reported [106].

#### 4.1.4. Mechanism

Regarding the mechanism of the cardiohemodynamic effects with S1P receptor modulators, a pre-clinical study suggested that S1P receptor subtype 1 (S1P1) in the heart could be one of the candidates for S1P receptor modulator-induced AV conduction block as subtype 5 is localised in the brain [107]. Initiation of fingolimod dosing triggers a decrease in HR and BP due to initial S1P1 receptor agonism, while continuous dosing results in the downregulation of S1P1 receptors, a subsequent shift in the S1P receptor profile and an increase in BP [108]. After an initial increase in parasympathetic regulation, continuous fingolimod dosing shifts cardiac autonomic regulation towards sympathetic predominance [65]. However, a recent observational study in 10 MS patients suggested that the first fingolimod dose may enhance the sympathetic heart modulation in addition to the expected parasympathetic activation in some people [73]. Another observational study in 21 MS patients suggested that fingolimod-associated HR slowing may result from vagomimetic fingolimod effects that start to wear off after approximately 4 h when the S1P receptor sensitivity to fingolimod begins to decrease and the receptor starts to internalise [74]. The authors speculate that the shift towards less sympathetic and more parasympathetic, or rather vagomimetic, cardiac modulation may be considered a beneficial effect in most patients, with only a small proportion being at risk of clinically relevant bradycardia or arrhythmias [74].

### 4.2. Other MS Drugs

While cardiac adverse effects (including ventricular arrhythmias, acute coronary syndromes, cardiomyopathies, AV conduction abnormalities, congestive heart failure and sudden cardiac death) of interferon alpha are well established, such effects have not been reported with interferon beta. A single case report of left bundle branch block in a patient treated with interferon beta-1a for MS has been published [109].

As part of the CLARITY Phase 3 trial of cladribine versus placebo in MS, an ECG sub-study evaluated potential acute and/or cumulative effects of cladribine on the ECG intervals (RR, PR, QRS, QT, QTcB and QTcF) and T-wave morphology [110]. This sub-study had a particular emphasis on the HR-corrected QT interval (QTcF, QTcB) as a well-accepted surrogate measure signifying delay or heterogeneity in cardiac ventricular repolarisation which may be associated with proarrhythmic characteristics of a drug. Cladribine showed no evidence of any effect on HR, AV conduction or cardiac depolarisation as measured by the PR and QRS interval durations. No significant changes in T-wave morphology were observed [110].

Finally, severe bradycardia has been reported as a rare adverse infusion-associated reaction with alemtuzumab [111].

Of note, but outside the scope of this review, treatment with mitoxantrone has been associated with cardiomyopathy, reduced left ventricular ejection fraction and (in 0.2%–0.5% of patients) congestive heart failure [112].

In addition to the findings with disease-modifying drugs discussed above, there have been some reports of adverse cardiac effects of corticosteroids. A study in 52 patients with acute MS relapse found that the most common cardiac arrhythmia before, during and after corticosteroid pulse therapy was sinus tachycardia; sinus bradycardia was observed in 42% of the recorded rhythms after pulse therapy [113]. There have also been case reports of nocturnal sinus bradycardia caused by intravenous methylprednisolone and symptomatic sinus bradycardia after treatment with high-dose oral prednisone in MS patients [114,115]. A recent case report describes a 27-year-old patient who developed atrial fibrillation on two occasions following two consecutive treatments with high-dose methylprednisolone for the treatment of MS relapse and was subsequently discovered to have mild sympathetic autonomic dysfunction [116].

Finally, it should be noted that a potential effect of other drugs on CAD in MS patients should not be dismissed. Polypharmacy is common in MS patients, as many suffer comorbidities. In one recent prospective study in 145 patients with RRMS in Germany, patients took on average 3.6 different medications including their MS drugs, with over 30% taking five or more medications. Some of the most common additional medications were analgesics (29% of patients), osteoporosis drugs (23%), contraceptives including hormone replacement therapy (22%), thyroid drugs (16%), antidepressants (12%), antihypertensives (12%) and gastrointestinal drugs (12%) [117].

## 5. Discussion

Substantial evidence shows that cardiac dysfunction and CAD is a common finding in people with MS. In the only meta-analysis on the subject, the prevalence of CAD was either 42% or 19%, depending on whether only one or at least two abnormal autonomic test results were used to define dysfunction [7]. The tests considered in this study were HRV to deep breathing, handgrip test, tilt test and Valsalva manoeuvre but no statement was made as to which of these tests should be combined. CAD may occur as a sudden symptom during a relapse and may respond positively to relapse treatment [85]. On the other hand, CAD can also occur insidiously during disease progression, but there is no data on whether disease-modifying therapy can prevent the occurrence of CAD [45,46,81].

A standardised recommendation for the detection of CAD in MS patients does not yet exist and it seems that testing for CAD may best be done using a combination of several tests, including short-term analysis of HRV. In fact, a wide range of tests have been used to screen for autonomic dysfunction, including time domain and frequency domain methods. While time domain methods screen for fluctuations of a signal and often employ distinct time points for evaluation, they often tend to omit a large part of the biosignal to be screened. Frequency domain methods, on the contrary, evaluate the distinct frequencies within the biosignal. Both techniques have a range of pitfalls to be aware of; in particular, frequency domain techniques are sensitive to artefacts and therefore need extremely careful and experienced method selection and data pre-screening. This review identifies an unmet need for a standard in autonomic evaluation in MS patients. While selected papers by themselves had a sound methodology, the techniques employed are divergent. Furthermore, not all possible dimensions of autonomic dysfunction are assessed in the reviewed literature, and it is generally methodologically difficult to attribute the reported findings to a peripheral or central cause, although a central cause is assumed in MS. In most studies, HRV is examined. HRV represents the spontaneous activity of cardiac autonomic function. Other parameters like heart rate recovery (HRR), which reflects the recovery of parasympathetic activity after maximal exercise or heart rate turbulence (HRT), which reflects the spontaneous response of baroreflex receptor activity to stress, were not studied in a standardised way [118]. In most studies, both the parasympathetic and sympathetic nervous systems are examined using the Ewing battery or parts of the Ewing battery. However, as described above, parameters or dimensions of autonomic dysfunction like HRR or HRT may be neglected. Therefore, we regard the evidence available at present as inappropriate for the derivation of structured recommendations. Table 1 shows the methods used so far to assess CAD. Impaired HRV is one of the most commonly reported findings indicating CAD in MS patients, and HR responses to the deep breathing test and the tilt-table test (30:15 ratio) are often lower in MS patients than in controls. Orthostatic intolerance/dizziness is a common finding (~50% of MS patients) and may result from impaired sympathetic vasoconstriction [77].

An important limitation in autonomic testing is the fact that autonomous measurement is subject to various factors, some of them very difficult to control. For example, there is the deconditioning of the patients, which is more or less pronounced depending on the degree of disability and is influenced by different training conditions even in patients with the same degree of disability [118]. Furthermore, age, patient medication, consumption of nicotine, alcoholics or caffeine or even room temperature in the examination room are important influencing factors in autonomic functional diagnostics. Therefore, strict standardization of patient preparation, test procedures and evaluation using standard values and test algorithms created for all laboratories is required [119].

CAD has been associated with lesions in the brain, especially the brainstem. However, the data are partly contradictory and the currently available data are not sufficient for a generally valid statement [2,17,43,46,47,48,50,53,64,80,85,86]. There is evidence that MS-related atrophy of the spinal cord correlates with CAD [46]. Since spinal affection is an independent predictor of MS-related disability, the question arises whether this is at least partially related to autonomic dysfunction. It therefore seems reasonable to perform MRI measurements of both the brain and the spine in future studies to gain further insight into the causes and effects of autonomic dysfunction in patients with MS. Furthermore, there is good evidence that CAD is worse in patients with progressive MS than in RRMS [45,46,81]. CAD appears to worsen with longer disease duration, although it may be present at diagnosis and worsen soon thereafter [41,49,51,52,53]. There is also some evidence for an association with EDSS and disease activity [5,43,76,82]. The association of CAD with fatigue in MS patients has been widely studied, but results are on the whole inconclusive. Relations between autonomic function, fatigue and cognition in MS patients are worth further study. Existing CAD may be a predictor of the well-documented first-dose cardiac effects of fingolimod [68,69,70,71,101], while rare cases of takotsubo cardiomyopathy in MS patients and life-threatening cardiac complications in those treated with fingolimod demonstrate the potential importance of CAD [13,14,19,20,21].

Our literature search also yielded data suggesting that balloon angioplasty may be useful in the treatment of autonomic dysfunction [61]. However, it must be considered that the concept of chronic cerebrospinal venous insufficiency has been disproved in the meantime and this intervention cannot be recommended according to the current state of knowledge [120].

## 6. Conclusions

Assessing CAD in MS patients may be important because of its correlation with underlying inflammation and neurodegeneration, its possible association with fatigue, rare serious cardiovascular complications and implications for treatment, especially with fingolimod. Overall, a substantial body of evidence suggests that patients with MS should be assessed for CAD. However, currently, a comparative superiority study on those techniques employed in MS patients is not available and a standard for autonomic evaluation in MS patients is lacking. However, it is worthwhile waiting for the results of the ongoing efforts of the European Federation of Autonomic Societies together with the American Autonomic Society to provide a consensus for cardiovascular autonomic testing. The publication of these consensus statements is expected. These have then to be evaluated and eventually applied to MS patients. No studies have yet looked at whether treating CAD can reduce the burden of MS symptoms, disease activity or the rate of progression.

## Figures and Tables

**Table 1:** Methods used so far to assess cardiac autonomic dysfunction.

Reference	Multiple Sclerosis (MS) Patients—*n*	MS Phenotype	Method	Time Domain Analysis	Frequency Domain Analysis	Results/Conclusion
Shirbani [4]	23	Not reported (NR)	Heart rate (HR), HR variability (HRV), blood pressure (BP), baroreflexsensitivity (BRS) at rest	x	x	compared to controls significantly lower baroreceptor sensitivity but higher systolic blood pressure variability in MS
Flachenecker [5]	26	Relapsing–remitting multiple sclerosis (RRMS)	Heart rate response (HRR), BP response (BPR), Ewing battery	x		parasympathetic dysfunction closely related to progression of disability, but sympathetic dysfunction associated with clinical activity of MS.
Merkelbach [8]	84	54 RRMS, 14 secondary progressive multiple sclerosis (SPMS), 16 primary progressive multiple sclerosis (PPMS)	HRR, BPR, Ewing battery	x		weakly significant correlation between autonomic tests and fatigue scores
Habek [17]	121	Clinically isolated syndrome (CIS)	HRR, BPR to Valsalva, deep breathing, tilt table	x		patients with CIS experienced worsening of ANS abnormalities during 2-year follow-up predicted by MRI
Krbot Skoric [18]	94	CIS	HRR, BPR to Valsalva, deep breathing, tilt table	x		three predictors for occurrence of new relapse: COMPASS-31 > 7.32, total number of T2 lesions > 3 and decreasing supine level of epinephrine
Hale [27]	31	NR	HRR, BPR to Valsalva, deep breathing, tilt table	x		abnormal autonomic function on laboratory testing in 5 MS patients, two of whom with abnormal heart rate response to the cycle test
Kodounis [26]	33	NR	HRV, BPR, QTc, Ewing battery	x		42.42% of the patients demonstrated ANS dysfunction
Videira [28]	20	RRMS	HRV, BPR, BRS Ewing battery with tilt test	x	x	HRV showed both parasympathetic and sympathetic impairment in MS
Diamond [29]	18	NR	HRV at rest	x	x	significantly lower vagal power in MS patients
Frontoni [30]	16	NR	HRV at rest and tilt-table, HRR, BPR, Ewing battery	x	x	no significant correlation between spectral ANS parameters and lesion area or localization as detected on MRI
Linden [31]	20	NR	HRR, BPR during deep breathing and tilt table	x	x	spectral and cross-spectral methods are valuable methods for evaluation of cardiovascular regulation
Brezinova [32]	36	29 RRMS, 4 SPMS, 3 PPMS	HRV during deep breathing and after active standing	x	x	spectral analysis of HRV in the rest-tilt-rest test differs significantly in MS patients compared to controls
Ferini-Strambi [33]	25	NR	HRR, BPR, RR interval variation during deep breathing, Valsalva, tilt table, HRV during sleep	x		A reduced parasympathetic activity in MS patients during both rapid eye movement and non-REM sleep
Keller [34]	11	RRMS	HR, BP at rest	x		reduced muscle sympathetic nerve activity and plasma norepinephrine in MS patients
Monge-Argiles [35]	34	NR	HRV 24-h electrocardiogram (ECG)	x	x	Increased sympathetic tone in MS patients as measured by HRV
Giubilei [36]	20	RRMS	HRR, BPR Ewing battery, HRV during sleep	x		HRV showed a lower degree of adaptability in MS patients
Tombul [37]	34	RRMS	HRV 24-h ECG	x		reduced HRV in MS patients
Ganz [38]	11	RRMS	HRR during deep breathing and mental stress	x	Lyapounov exponent	significant lower Lyapounov exponent suggesting less adaptive central autonomic organisation
Habek [39]	104	CIS	HRV, HRR, BPR to Valsalva, deep breathing, passive tilt	x	x	Autonomic dysfunction in 59.8% of patients, parasympathetic dysfunction in 5%, sympathetic in 42.6%
Al Araji [40]	55	NR	HRR, BPR, Ewing battery	x		Autonomic symptoms significantly more prevalent in MS patients than in controls
Gunal [41]	22	RRMS	HRR, BPR, Ewing battery	x		90% of the patients had symptoms related to autonomic dysfunction, 45.5% had abnormal cardiovascular autonomic function testing
Thomaides [42]	10	SPMS	HRR, BPR during handgrip, Valsalva, deep breathing, tilt and mental stress	x		impaired pressor responses in disabled patients
Saari [43]	51	NR	HRR, BPR during normal breathing, Ewing battery with tilt table test	x		decreased HRV during tilt table test and deep breathing in MS patients
Gervasoni [44]	23	NR	HRV at rest, standing, light exercise and recovery	x	x	in MS patients HRV slightly but not significantly higher, significantly lower parasympathetic indexes during baseline and post-exercise
Adamec [45]	70	40 RRMS, 30 progressive multiple sclerosis (PMS)	HRR and BPR to Valsalva, HRR to deep breathing, BPR to tilt table	x	x	significantly higher total CASS score in PMS patients compared to RRMS
Studer [45]	120	84 RRMS, 36 PMS	HRV during rest and tilt table test	x	x	sympathetic dysfunction as measured by HRV closely related to the progression of disability in MS patients
de Seze [46]	75	25 RRMS, 25 SPMS, 25 PPMS	HRV, BPR during deep breathing, Valsalva, standing up test	x		correlation of autonomic dysfunction with spinal cord atrophy
Vita [47]	40	NR	HRV, BPR, R-R interval variation at rest and Ewing battery	x		significant association between presence of autonomic dysfunction and clinical and MRI evidence of brainstem lesions
Brinar [48]	28	RRMS	HRR during Valsalva and cortical activation, HRR and BPR during quick standing,	x		positive correlation between autonomic dysfunction and MRI findings
Mahovic [49]	39	RRMS	HRV 24-h ECG	x	x	significantly lower HRV in MS patients than in controls
Damla [50]	51	RRMS	HRV 24-h ECG	x	x	decreased HRV in MS compared to controls, but no correlation with disability or disease activity
Vlcek [51]	19	RRMS	HRV during Stroop test	x	x	lower HR and lower epinephrine increment after Stroop test in MS patients compared to controls
Nasseri [52]	46	20 RRMS, 26 SPMS	HRR during deep breathing, standing up test and Valsalva	x		progression of autonomic dysfunction over 1 year
Nasseri [53]	20	RRMS	HRR during deep breathing, standing up test and Valsalva	x		progression of autonomic dysfunction over 2 years without significant progression of EDSS
Krbot Skoric [54]	70	NR	HRR and BPR to Valsalva and Tilt table, HRV to deep breathing	x		clear association between fatigue and scores in subjective tests but only modest correlation between fatigue and objective test of the ANS
Lebre [55]	50	RRMS	HRV, BPR, Ewing battery	x		reduced capacity to increase blood pressure in MS patients with fatigue
Flachenecker [56]	60	NR	HRV, BPR, Ewing battery	x	x	autonomic responses correlated with fatigue
Keselbrenner [57]	10	NR	HRV, BPR at rest and standing	x	x	age-related reduction in vagal activity occurred earlier in patients with MS who experienced fatigue
Heesen [58]	23	19 RRMS, 3 SPMS, 1 RPMS	HRV during stress	x		cognitive stress induces IFNg production in healthy controls but not in MS patients with fatigue, reduced cardiac response in MS patients
Sander [59]	53	36 RRMS, 10 SPMS, 7 PPMS	HRV during vigilance task	x	x	Cognitive fatigue in MS is related to parasympathetic activity
Niepel [60]	26	21 RRMS, 3 SPMS, 2 PPMS	HR and BP in sitting position and during handgrip before and after modafinil	x		MS patients with fatigue had a reduced level of cardiovascular sympathetic activation
Arata [61]	21	10 RRMS, 5 SPMS, 6 PPMS	HRV during deep breathing, Valsalva and after active standing	x		improvement of autonomic dysfunction after balloon angioplasty of cerebral veins
Huang [62]	10	RRMS	BPR and HRR to carotid baroreflex stimulation	x		impaired baroreflex control of blood pressure in MS patients
Sanya [63]	13	RRMS	BPR and HRR to carotid baroreflex stimulation	x	x	baroreflex dysfunction in MS patients
Winder [64]	74	RRMS	HRV, BPR at rest	x	x	association between increased sympathetic modulation and left insular and hippocampal lesions
Simula [65]	27	RRMS	HRV 24-h ECG	x	x	fingolimod dosing shifts cardiac autonomic regulation towards sympathetic predominance
Vehoff [66]	33	RRMS	HRV, BPR during breathing at rest, deep breathing, Valsalva before and after fingolimod	x		initial increase in HRV, measured 4.5 h after first intake of fingolimod, but substantial decrease in HRV within next 3 months
Akbulak [67]	64	RRMS	HRV 24-h ECG	x	x	decreased HRV up to 72 h after fingolimod intake
Rossi [68]	55	RRMS	HRV, BPR Ewing battery with tilt table	x	x	significant correlations between measures of parasympathetic function and fingolimod-induced bradycardia
Li [69]	78	RRMS	HRV at rest before and after Fingolimod	x	x	increase of HRV parameters representing parasympathetic activities two hours after fingolimod administration
Vanoli [70]	625	RRMS	HRV at rest before and after Fingolimod	x	x	normalized spectral power in the high-frequency band and previous annualized relapse rate were independently correlated with the probability of undergoing extended monitoring
Hilz [71]	21	RRMS	HRV, BPR, Ewing battery	x	x	patients with higher resting BP and higher BP increase during handgrip-exercise had prolonged HR slowing after Fingolimod
Tran [72]	113	RRMS	HRV 24-h ECG	x	x	ozanimod does not prolong QTc interval
Racca [73]	10	RRMS	HRV, BP after baroreflex stimulation	x	x	no modification of baroreflex sensitivity after first dose of Fingolimod
Hilz [74]	21	RRMS	HRV, BP at rest before and after fingolimod	x	x	increases in parasympathetic and cardiac autonomic modulation after fingolimod

**Table 2:** Cardiac autonomic dysfunction in the Phase 3 trials of fingolimod [88,89].

	FREEDOMS			TRANSFORMS		
	Fingolimod		Placebo (*n* = 418)	Fingolimod		Placebo (*n* = 431)
	1.25 mg (*n*= 429)	0.5 mg (*n* = 425)		1.25 mg (*n*= 420)	0.5 mg (*n* = 429)	
**Cardiovascular adverse events—n (%)**						
Hypertension	27 (6.3)	26 (6.1)	16 (3.8)	21 (5.0)	16 (3.7)	8 (1.9)
Bradycardia, bradyarrhythmia, or sinus bradycardia	14 (3.3)	9 (2.1)	3 (0.7)	10 (2.4)	2 (0.5)	0
First degree atrioventricular (AV) block	5 (1.2)	2 (0.5)	2 (0.5)	3 (0.7)	1 (0.2)	0
Second degree AV block	1 (0.2)	0	1 (0.2)	2 (0.5)	1 (0.2)	0
**Cardiac monitoring**						
Maximum first-dose heart rate decrease—bpm	10	8	–	12	8	–
First degree AV block—*n* (%)	37 (8.6)	20 (4.7)	6 (1.4)	–	–	–
Second degree AV block—*n* (%)	4 (0.9)	1 (0.2)	0	–	–	–
Mean blood pressure increase in first 6 months	3.6/2.1 mmHg	1.9/0.7 mmHg	–	3 mmHg	2 mmHg	–

**Table 3:** Cardiac monitoring results in the FREEDOMS II study [91].

	Fingolimod		Placebo (*n* = 355)
	1.25 mg (*n* = 370)	0.5 mg (*n* = 358)	
**First dose monitoring events**			
Bradycardia	21 (6%)	5 (1%)	1 (<0.5%)
Symptomatic bradycardia	15 (4%)	3 (1%)	1 (<0.5%)
First-degree atrioventricular (AV) block	35 (10%)	17 (5%)	7 (2%)
Mobitz I AV block	6 (2%)	0	0
Second-degree AV block	1 (<0.5%)	0	0
**24-h Holter monitoring events**			
**First dose**			
Mobitz I (Wenckebach) second-degree AV block	24 (7%)	13 (4%)	7 (2%)
Second-degree AV block	12 (3%)	7 (2%)	0
**3 months**			
Mobitz I (Wenckebach) second-degree AV block	0	0	5 (2%)
Second-degree AV block	0	0	1 (<0.5%)

## Supplementary Materials

The following are available online at https://www.mdpi.com/2077-0383/9/2/335/s1, File S1. Articles excluded after full text review.

## References

[1] Racosta J.M., Kimpinski K., Morrow S.A., Kremenchutzky M. (2015). Autonomic dysfunction in multiple sclerosis. Auton. Neurosci..

[2] Valencia-Sanchez C., Goodman B.P., Carter J.L., Wingerchuk D.M. (2019). The spectrum of acute cardiopulmonary events associated with multiple sclerosis exacerbations. Mult. Scler. J..

[3] Racosta J.M., Kimpinski K. (2016). Autonomic dysfunction, immune regulation, and multiple sclerosis. Clin. Auton. Res..

[4] Shirbani F., Barin E., Lee Y.-C., Ng K., Parratt J.D.E., Butlin M., Avolio A.P. (2018). Characterisation of cardiac autonomic function in multiple sclerosis based on spontaneous changes of heart rate and blood pressure. Mult. Scler. Relat. Disord..

[5] Flachenecker P., Reiners K., Krauser M., Wolf A., Toyka K.V. (2001). Autonomic dysfunction in multiple sclerosis is related to disease activity and progression of disability. Mult. Scler. J..

[6] Habek M. (2019). Immune and autonomic nervous system interactions in multiple sclerosis: Clinical implications. Clin. Auton. Res..

[7] Racosta J.M., Sposato L.A., Morrow S.A., Cipriano L.E., Kimpiski K., Kremenchutzky M. (2015). Cardiovascular autonomic dysfunction in multiple sclerosis: A meta-analysis. Mult. Scler. Relat. Disord..

[8] Merkelbach S., Dillmann U., Kölmel C., Holz J., Müller M. (2001). Cardiovascular autonomic dysregulation and fatigue in multiple sclerosis. Mult. Scler. J..

[9] Christiansen C.F., Christensen S., Farkas D.K., Miret M., Sørensen H.T., Pedersen L. (2010). Risk of Arterial Cardiovascular Diseases in Patients with Multiple Sclerosis: A Population-Based Cohort Study. Neuroepidemiology.

[10] Kaplan T.B., Berkowitz A.L., Samuels M.A. (2015). Cardiovascular Dysfunction in Multiple Sclerosis. The Neurologist.

[11] Scalfari A., Knappertz V., Cutter G., Goodin D.S., Ashton R., Ebers G.C. (2013). Mortality in patients with multiple sclerosis. Neurology.

[12] Hilz M.J. (2016). Cardiac stunning as first manifestation of multiple sclerosis: A case report reminding us not to overlook cardiovascular autonomic dysfunction in multiple sclerosis. Mult. Scler. J..

[13] Midaglia L., Juega Mariño J.M., Sastre-Garriga J., Rovira A., Vidal-Jordana A., López-Pérez M.A., Montalban X. (2016). An uncommon first manifestation of multiple sclerosis: Tako-Tsubo cardiomyopathy. Mult. Scler. J..

[14] London F., Gonzalez Rodriguez de Azero N., Philippart M., Higny J., Mulquin N. (2019). Reverse takotsubo cardiomyopathy triggered by a multiple sclerosis relapse. Acta Neurol. Belg..

[15] Madias J.E. (2019). Takotsubo syndrome triggered in the setting of multiple sclerosis: The need to monitor blood catecholamines and the CNS sympathetic input to the heart. Mult. Scler. Relat. Disord..

[16] Bayer A.D., Cahill J.F., Rizvi S.A. (2019). Multiple sclerosis relapse presenting as an acute cardiomyopathy. Mult. Scler. Relat. Disord..

[17] Habek M., Crnošija L., Gabelić T., Barun B., Adamec I., Junaković A., Ruška B., Pavičić T., Skorić M.K. (2019). Longitudinal assessment of autonomic nervous system in patients with first demyelinating event suggestive of multiple sclerosis. Eur. J. Neurol..

[18] Skorić M.K., Crnošija L., Gabelić T., Barun B., Adamec I., Junaković A., Pavičić T., Ruška B., Habek M. (2019). Autonomic symptom burden can predict disease activity in early multiple sclerosis. Mult. Scler. Relat. Disord..

[19] Rosini J.M., Rajasimhan S., Fellows S.E., Nomura J.T. (2015). Delayed cardiac dysrhythmias after fingolimod administration. Am. J. Emerg. Med..

[20] Espinosa P.S., Berger J.R. (2011). Delayed fingolimod-associated asystole. Mult. Scler. J..

[21] Lindsey J., Haden-Pinneri K., Memon N., Buja L.M. (2012). Sudden unexpected death on fingolimod. Mult. Scler. J..

[22] Feige J., Schernthaner C., Wipfler P., Sellner J. (2020). Delayed high-grade atrioventricular block requiring pacemaker implantation in a multiple sclerosis patient treated with fingolimod. Mult. Scler. Relat. Disord..

[23] Koçer A., Karakaya O., Kargin R., Barutcu I., Esen A.M. (2005). P wave duration and dispersion in multiple sclerosis. Clin. Auton. Res..

[24] Razazian N., Hedayati N., Moradian N., Bostani A., Afshari D., Asgari N. (2014). P wave duration and dispersion and QT interval in multiple sclerosis. Mult. Scler. Relat. Disord..

[25] Turri G., Calabrese M., Pancheri E., Monaco S., Gajofatto A., Marafioti V. (2017). QTc interval in patients with multiple sclerosis: An inference from the insula of Reil?. Eur. J. Neurol..

[26] Kodounis A., Stamboulis E., Constantinidis T.S., Liolios A. (2005). Measurement of autonomic dysregulation in multiple sclerosis. Acta Neurol. Scand..

[27] Hale L.A., Nukada H., Du Plessis L.J., Peebles K.C. (2009). Clinical screening of autonomic dysfunction in multiple sclerosis. Physiother. Res. Int..

[28] Videira G., Castro P., Vieira B., Filipe J.P., Santos R., Azevedo E., Sá M.J., Abreu P. (2016). Autonomic dysfunction in multiple sclerosis is better detected by heart rate variability and is not correlated with central autonomic network damage. J. Neurol. Sci..

[29] Diamond B., Kim H., DeLuca J., Cordero D. (1995). Cardiovascular regulation in multiple sclerosis. Mult. Scler. J..

[30] Frontoni M., Fiorini M., Strano S., Cerutti S., Giubilei F., Urani C., Bastianello S., Pozzilli C. (1996). Power spectrum analysis contribution to the detection of cardiovascular dysautonomia in multiple sclerosis. Acta Neurol. Scand..

[31] Linden D., Diehl R.R., Kretzschmar A., Berlit P. (1997). Autonomic evaluation by means of standard tests and power spectral analysis in multiple sclerosis. Muscle Nerve.

[32] Brezinova M., Goldenberg Z., Kucera P. (2004). Autonomic nervous system dysfunction in multiple sclerosis patients. Bratisl. Lek. Listy.

[33] Ferini-Strambi L., Rovaris M., Oldani A., Martinelli V., Filippi M., Smirne S., Zucconi M., Comi G. (1995). Cardiac autonomic function during sleep and wakefulness in multiple sclerosis. J. Neurol..

[34] Keller D.M., Fadel P.J., Harnsberger M.A., Remington G.M., Frohman E.M., Davis S.L. (2014). Reduced spontaneous sympathetic nerve activity in multiple sclerosis patients. J. Neurol. Sci..

[35] Monge A.J.A., Palacios O.F., Vila-Sobrino J.A., Matias-Guiu J. (1998). Heart rate variability in multiple sclerosis during a stable phase. Acta Neurol. Scand..

[36] Giubilei F., Vitale A., Urani C., Fronton M., Fiorini M., Millefiorini E., Fiorelli M., Santini M., Strano S., Frontoni M. (1996). Cardiac Autonomic Dysfunction in Relapsing-Remitting Multiple Sclerosis during a Stable Phase. Eur. Neurol..

[37] Tombul T., Anlar O., Tuncer M., Huseyinoglu N., Eryonucu B. (2011). Impaired heart rate variability as a marker of cardiovascular autonomic dysfunction in multiple sclerosis. Acta Neurol. Belg..

[38] Ganz R.E., Weibels G., Stäcker K.-H., Faustmann P.M., Zimmermann C.W. (1993). The Lyapunov Exponent of Heart Rate Dynamics as a Sensitive Marker of Central Autonomic Organization: An Exemplary Study of Early Multiple Sclerosis. Int. J. Neurosci..

[39] Habek M., Crnošija L., Lovrić M., Junaković A., Skorić M.K., Adamec I. (2016). Sympathetic cardiovascular and sudomotor functions are frequently affected in early multiple sclerosis. Clin. Auton. Res..

[40] Al-Araji A.H., Al-Mahdawi A.M., Mohammad A.I. (2003). Autonomic dysfunction in multiple sclerosis. Neurosciences.

[41] Gunal D.I., Afsar N., Tanridag T., Aktan S. (2002). Autonomic dysfunction in multiple sclerosis: Correlation with disease-related parameters. Eur. Neurol..

[42] Thomaides T.N., Zoukos Y., Chaudhuri K.R., Mathias C.J. (1993). Physiological assessment of aspects of autonomic function in patients with secondary progressive multiple sclerosis. J. Neurol..

[43] Saari A., Tolonen U., Pääkkö E., Suominen K., Pyhtinen J., Sotaniemi K., Myllyla V. (2004). Cardiovascular autonomic dysfunction correlates with brain MRI lesion load in MS. Clin. Neurophysiol..

[44] Gervasoni E., Bove M., Sinatra M., Grosso C., Rovaris M., Cattaneo D., Merati G. (2018). Cardiac autonomic function during postural changes and exercise in people with multiple sclerosis: A cross-sectional study. Mult. Scler. Relat. Disord..

[45] Adamec I., Crnošija L., Junaković A., Skorić M.K., Habek M. (2018). Progressive multiple sclerosis patients have a higher burden of autonomic dysfunction compared to relapsing remitting phenotype. Clin. Neurophysiol..

[46] De Seze J., Stojkovic T., Gauvrit J.-Y., Devos D., Ayachi M., Cassim F., Michel T.S., Pruvo J.-P., Guieu J.-D., Vermersch P. (2001). Autonomic dysfunction in multiple sclerosis: Cervical spinal cord atrophy correlates. J. Neurol..

[47] Vita G., Fazio M.C., Milone S., Blandino A., Salvi L., Messina C. (1993). Cardiovascular autonomic dysfunction in multiple sclerosis is likely related to brainstem lesions. J. Neurol. Sci..

[48] Brinar V., Brzović Z., Papa J., Malojcić B., Dawidowsky K. (1997). Autonomic dysfunction in patients with multiple sclerosis. Coll. Antropol..

[49] Mahovic D., Lakusic N. (2007). Progressive Impairment of Autonomic Control of Heart Rate in Patients with Multiple Sclerosis. Arch. Med. Res..

[50] Damla O., Altug C., Pinar K.K., Alper K., Dilek I.G., Kadriye A. (2018). Heart rate variability analysis in patients with multiple sclerosis. Mult. Scler. Relat. Disord..

[51] Vlcek M., Penesova A., Imrich R., Meskova M., Mravcova M., Grunnerova L., Garafova A., Sivakova M., Turcani P., Kollar B. (2018). Autonomic Nervous System Response to Stressors in Newly Diagnosed Patients with Multiple Sclerosis. Cell. Mol. Neurobiol..

[52] Nasseri K., TenVoorde B.J., Adèr H.J., Uitdehaag B., Polman C.H. (1998). Longitudinal follow-up of cardiovascular reflex tests in multiple sclerosis. J. Neurol. Sci..

[53] Nasseri K., Uitdehaag B.M.J., Walderveen M.A., Adèr H.J., Polman C.H. (1999). Cardiovascular autonomic function in patients with relapsing remitting multiple sclerosis: A new surrogate marker of disease evolution?. Eur. J. Neurol..

[54] Krbot S.M., Crnosija L., Adamec I., Barun B., Gabelic T., Smoljo T., Stanic I., Pavicic T., Pavlovic I., Drulovic J. (2019). Autonomic symptom burden is an independent contributor to multiple sclerosis related fatigue. Clin. Auton. Res..

[55] Lebre A.T., Mendes M.F., Tilbery C.P., Almeida A.L., Neto A.S. (2007). Relation between fatigue and autonomic disturbances in multiple sclerosis. Arq. Neuro Psiquiatr..

[56] Flachenecker P., Rufer A., Bihler I., Hippel C., Reiners K., Toyka K., Kesselring J. (2003). Fatigue in MS is related to sympathetic vasomotor dysfunction. Neurology.

[57] Keselbrener L., Akselrod S., Ahiron A., Eldar M., Barak Y., Rotstein Z. (2000). Is fatigue in patients with multiple sclerosis related to autonomic dysfunction?. Clin. Auton. Res..

[58] Heesen C., Koehler G., Gross R., Tessmer W., Schulz K.-H., Gold S.M. (2005). Altered cytokine responses to cognitive stress in multiple sclerosis patients with fatigue. Mult. Scler. J..

[59] Sander C., Modes F., Schlake H.-P., Eling P., Hildebrandt H. (2019). Capturing fatigue parameters: The impact of vagal processing in multiple sclerosis related cognitive fatigue. Mult. Scler. Relat. Disord..

[60] Niepel G., Bibani R.H., Vilisaar J., Langley R.W., Bradshaw C.M., Szabadi E., Constantinescu C.S. (2013). Association of a deficit of arousal with fatigue in multiple sclerosis: Effect of modafinil. Neuropharmacology.

[61] Arata M., Sternberg Z. (2014). Transvascular Autonomic Modulation: A Modified Balloon Angioplasty Technique for the Treatment of Autonomic Dysfunction in Multiple Sclerosis Patients. J. Endovasc. Ther..

[62] Huang M., Allen D.R., Keller D.M., Fadel P.J., Frohman E.M., Davis S.L. (2016). Impaired carotid baroreflex control of arterial blood pressure in multiple sclerosis. J. Neurophysiol..

[63] Sanya E.O., Tutaj M., Brown C.M., Goel N., Neundörfer B., Hilz M.J., Sanya E.O. (2005). Abnormal heart rate and blood pressure responses to baroreflex stimulation in multiple sclerosis patients. Clin. Auton. Res..

[64] Winder K., Linker R.A., Seifert F., Wang R., Lee D., Engelhorn T., Dörfler A., Fröhlich K., Hilz M. (2019). Cerebral lesion correlates of sympathetic cardiovascular activation in multiple sclerosis. Hum. Brain Mapp..

[65] Simula S., Laitinen T., Laitinen T.M., Tarkiainen T., Hartikainen P., Hartikainen J.E. (2016). Effect of fingolimod on cardiac autonomic regulation in patients with multiple sclerosis. Mult. Scler. J..

[66] Vehoff J., Haegele-Link S., Humm A., Kaegi G., Mueller S.K., Sauter R., Tettenborn B.E., Hundsberger T. (2017). Heart rate variability decreases after 3 months of sustained treatment with fingolimod. J. Neurol..

[67] Akbulak R.Ö., Rosenkranz S.C., Schaeffer B.N., Pinnschmidt H.O., Willems S., Heesen C., Hoffmann B.A. (2017). Acute and long-term effects of fingolimod on heart rhythm and heart rate variability in patients with multiple sclerosis. Mult. Scler. Relat. Disord..

[68] Rossi S., Rocchi C., Studer V., Motta C., Lauretti B., Germani G., Macchiarulo G., Marfia G.A., Centonze D. (2015). The autonomic balance predicts cardiac responses after the first dose of fingolimod. Mult. Scler. J..

[69] Li K., Konofalska U., Akgün K., Reimann M., Rüdiger H., Haase R., Ziemssen T. (2017). Modulation of Cardiac Autonomic Function by Fingolimod Initiation and Predictors for Fingolimod Induced Bradycardia in Patients with Multiple Sclerosis. Front. Mol. Neurosci..

[70] Vanoli E., Montano N., De Angelis G., Badilini F., Mirabella M., Bonavita S., Patti F., Bianco A., Sparaco M., Chisari C. (2019). Cardiovascular autonomic individual profile of relapsing-remitting multiple sclerosis patients and risk of extending cardiac monitoring after first dose fingolimod. J. Neurol. Sci..

[71] Hilz M.J., Intravooth T., Moeller S., Wang R., Lee D.-H., Koehn J., Linker R.A. (2015). Central Autonomic Dysfunction Delays Recovery of Fingolimod Induced Heart Rate Slowing. PLoS ONE.

[72] Tran J.Q., Hartung J.P., Olson A.D., Mendzelevski B., Timony G.A., Boehm M.F., Peach R.J., Gujrathi S., Frohna P.A. (2018). Cardiac Safety of Ozanimod, a Novel Sphingosine-1-Phosphate Receptor Modulator: Results of a Thorough QT/QTc Study. Clin. Pharmacol. Drug Dev..

[73] Racca V., Rovaris M., Cavarretta R., Vaini E., Toccafondi A., Di Rienzo M. (2019). Acute Fingolimod Effects on Baroreflex and Cardiovascular Autonomic Control in Multiple Sclerosis. J. Cent. Nerv. Syst. Dis..

[74] Hilz M.J., Wang R., Leal C.D.R., Liu M., Canavese F., Roy S., Hösl K.M., Winder K., Lee D.-H., Linker R.A. (2017). Fingolimod initiation in multiple sclerosis patients is associated with potential beneficial cardiovascular autonomic effects. Ther. Adv. Neurol. Disord..

[75] Olindo S., Guillon B., Helias J., Phillibert B., Magne C., Feve J.R. (2002). Decrease in heart ventricular ejection fraction during multiple sclerosis. Eur. J. Neurol..

[76] Kale N., Magana S., Agaoglu J., Tanik O., Minelli C., Gondim F.A., Barreira A.A., Dromerick A.W. (2009). Assessment of autonomic nervous system dysfunction in multiple sclerosis and the association with clinical disability. Neurol. Int..

[77] Flachenecker P., Wolf A., Krauser M., Hartung H.-P., Reiners K. (1999). Cardiovascular autonomic dysfunction in multiple sclerosis: Correlation with orthostatic intolerance. J. Neurol..

[78] Kanjwal K. (2010). Autonomic Dysfunction Presenting as Postural Orthostatic Tachycardia Syndrome in Patients with Multiple Sclerosis. Int. J. Med. Sci..

[79] Ibrahim M., Saint-Croix G., Colombo R. (2019). Brugada Syndrome Caused by Autonomic Dysfunction in Multiple Sclerosis. Case Rep. Cardiol..

[80] Jurić S., Mišmaš A., Mihić N., Barać A.M., Habek M. (2012). Newly onset sinus bradycardia in the context of multiple sclerosis relapse. Intern. Med..

[81] Studer V., Rocchi C., Motta C., Lauretti B., Perugini J., Brambilla L., Centonze D. (2017). Heart rate variability is differentially altered in multiple sclerosis: Implications for acute, worsening and progressive disability. Mult. Scler. J. Exp. Transl. Clin..

[82] Ziqber J., Chmielewski H., Dryjariski T., Goch J.H. (1997). Evaluation of myocardial muscle functional parameters in patients with multiple sclerosis. Acta Neurol. Scand..

[83] Mikkola A., Ojanen A., Hartikainen J.E.K., Remes A.M., Simula S. (2017). The impact of multiple sclerosis onset symptom on cardiac repolarization. Brain Behav..

[84] Mikkola A., Ojanen A., Hartikainen J.E., Remes A.M., Simula S. (2018). Cardiac repolarization evolves differently during the course of benign and disabling multiple sclerosis. Mult. Scler. Relat. Disord..

[85] Sakakibara R., Mori M., Fukutake T., Kita K., Hattori T. (1997). Orthostatic hypotension in a case with multiple sclerosis. Clin. Auton. Res..

[86] Schroth W.S., Tenner S.M., Rappaport B.A., Mani R. (1992). Multiple Sclerosis as a Cause of Atrial Fibrillation and Electrocardiographic Changes. Arch. Neurol..

[87] Kappos L., Antel J., Comi G., Montalban X., O’Connor P., Polman C.H., Haas T., Korn A.A., Karlsson G., Radue E.W. (2006). Oral Fingolimod (FTY720) for Relapsing Multiple Sclerosis. N. Engl. J. Med..

[88] Kappos L., Radue E.-W., O’Connor P., Polman C., Hohlfeld R., Calabresi P., Selmaj K., Agoropoulou C., Leyk M., Zhang-Auberson L. (2010). A Placebo-Controlled Trial of Oral Fingolimod in Relapsing Multiple Sclerosis. N. Engl. J. Med..

[89] Cohen J.A., Barkhof F., Comi G., Hartung H.-P., Khatri B.O., Montalban X., Pelletier J., Capra R., Gallo P., Izquierdo G. (2010). Oral Fingolimod or Intramuscular Interferon for Relapsing Multiple Sclerosis. N. Engl. J. Med..

[90] Kappos L., O’Connor P., Radue E.-W., Polman C., Hohlfeld R., Selmaj K., Ritter S., Schlosshauer R., Von Rosenstiel P., Zhang-Auberson L. (2015). Long-term effects of fingolimod in multiple sclerosis: The randomized FREEDOMS extension trial. Neurology.

[91] Calabresi P.A., Radue E.-W., Goodin U., Jeffery U., Rammohan K.W., Reder A.T., Vollmer T., Agius M.A., Kappos L., Stites T. (2014). Safety and efficacy of fingolimod in patients with relapsing-remitting multiple sclerosis (FREEDOMS II): A double-blind, randomised, placebo-controlled, phase 3 trial. Lancet Neurol..

[92] Kappos L., Cohen J., Collins W., De Vera A., Zhang-Auberson L., Ritter S., Von Rosenstiel P., Francis G. (2014). Fingolimod in relapsing multiple sclerosis: An integrated analysis of safety findings. Mult. Scler. Relat. Disord..

[93] DiMarco J.P., O’Connor P., Cohen J.A., Reder A.T., Zhang-Auberson L., Tang D., Collins W., Kappos L. (2014). First-dose effects of fingolimod: Pooled safety data from three phase 3 studies. Mult. Scler. Relat. Disord..

[94] Gold R., Comi G., Palace J., Siever A., Gottschalk R., Bijarnia M., von Rosenstiel P., Tomic D., Kappos L. (2014). Assessment of cardiac safety during fingolimod treatment initiation in a real-world relapsing multiple sclerosis population: A phase 3b, open-label study. J. Neurol..

[95] Laroni A., Brogi D., Morra V.B., Guidi L., Pozzilli C., Comi G., Lugaresi A., Turrini R., Raimondi D., Uccelli A. (2014). Safety of the first dose of fingolimod for multiple sclerosis: Results of an open-label clinical trial. BMC Neurol..

[96] Limmroth V., Ziemssen T., Lang M., Richter S., Wagner B., Haas J., Schmidt S., Gerbershagen K., Lassek C., Klotz L. (2017). Electrocardiographic assessments and cardiac events after fingolimod first dose—A comprehensive monitoring study. BMC Neurol..

[97] Ontaneda D., Hara-Cleaver C., Rudick R.A., Cohen J.A., Bermel R.A. (2012). Early tolerability and safety of fingolimod in clinical practice. J. Neurol. Sci..

[98] Fragoso Y.D., Arruda C.C., Arruda W.O., Brooks J.B.B., Damasceno A., Damasceno C.A.D.A., Finkelsztejn A., Finkelsztejn J., Da Gama P.D., Giacomo M.C.B. (2014). The real-life experience with cardiovascular complications in the first dose of fingolimod for multiple sclerosis. Arq. Neuro Psiquiatr..

[99] Paolicelli D., Manni A., DiRenzo V., D’Onghia M., Tortorella C., Zoccolella S., Trojano M. (2015). Long term cardiac safety and tolerability of Fingolimod in Multiple Sclerosis: A post-marketing study. J. Clin. Pharmacol..

[100] Castillo-Trivino T., Lopetegui I., De Munain A.L., Olascoaga J., Alarcón-Duque J.A. (2015). Ventricular tachycardia on chronic fingolimod treatment for multiple sclerosis. J. Neurol. Neurosurg. Psychiatry.

[101] Kocyigit D., Yalcin M.U., Gurses K.M., Tokgozoglu L., Karabudak R. (2019). Are there any clinical and electrocardiographic predictors of heart rate reduction in relapsing- remitting multiple sclerosis patients treated with fingolimod?. Mult. Scler. Relat. Disord..

[102] Selmaj K., Li D.K., Hartung H.-P., Hemmer B., Kappos L., Freedman M.S., Stuve O., Rieckmann P., Montalban X., Ziemssen T. (2013). Siponimod for patients with relapsing-remitting multiple sclerosis (BOLD): An adaptive, dose-ranging, randomised, phase 2 study. Lancet Neurol..

[103] Kappos L., Li D.K.B., Stüve O., Hartung H.-P., Freedman M.S., Hemmer B., Rieckmann P., Montalban X., Ziemssen T., Hunter B. (2016). Safety and Efficacy of Siponimod (BAF312) in Patients With Relapsing-Remitting Multiple Sclerosis. JAMA Neurol..

[104] Kappos L., Bar-Or A., Cree B.A.C., Fox R.J., Giovannoni G., Gold R., Vermersch P., Arnold D.L., Arnould S., Scherz T. (2018). Siponimod versus placebo in secondary progressive multiple sclerosis (EXPAND): A double-blind, randomised, phase 3 study. Lancet.

[105] Cohen J.A., Arnold D.L., Comi G., Bar-Or A., Gujrathi S., Hartung J.P., Cravets M., Olson A., Frohna P.A., Selmaj K.W. (2016). Safety and efficacy of the selective sphingosine 1-phosphate receptor modulator ozanimod in relapsing multiple sclerosis (RADIANCE): A randomised, placebo-controlled, phase 2 trial. Lancet Neurol..

[106] Comi G., Kappos L., Selmaj K.W., Bar-Or A., Arnold D.L., Steinman L., Hartung H.-P., Montalban X., Havrdová E.K., Cree B.A.C. (2019). Safety and efficacy of ozanimod versus interferon beta-1a in relapsing multiple sclerosis (SUNBEAM): A multicentre, randomised, minimum 12-month, phase 3 trial. Lancet Neurol..

[107] Yagi Y., Nakamura Y., Kitahara K., Harada T., Kato K., Ninomiya T., Cao X., Ohara H., Izumi-Nakaseko H., Suzuki K. (2014). Analysis of Onset Mechanisms of a Sphingosine 1-Phosphate Receptor Modulator Fingolimod-Induced Atrioventricular Conduction Block and QT-Interval Prolongation. Toxicol. Appl. Pharmacol..

[108] Camm J., Hla T., Bakshi R., Brinkmann V. (2014). Cardiac and vascular effects of fingolimod: Mechanistic basis and clinical implications. Am. Hear. J..

[109] Kastalli S., El Aidli S., Mourali S., Zaiem A., Daghfous R., Lakhal M. (2012). Cardiac arrhythmia induced by interferon beta-1a. Fundam. Clin. Pharm..

[110] Hermann R., Litwin J.S., Friberg L.E., Dangond F., Munafo A. (2019). Effects of cladribine tablets on heart rate, atrio-ventricular conduction and cardiac repolarization in patients with relapsing multiple sclerosis. Br. J. Clin. Pharm..

[111] Nerrant E., Thouvenot E., Castelnovo G. (2017). Severe bradycardia: An unreported adverse infusion-associated reaction (IAR) with alemtuzumab. Rev. Neurol..

[112] Pastuszak Z., Tomczykiewicz K., Piusinska-Macoch R., Stepien A. (2016). Cardiac effects of mitoxanthrone therapy in patients with multiple sclerosis. Kardiol. Pol..

[113] Vasheghani-Farahani A., Sahraian M.A., Darabi L., Aghsaie A., Minagar A. (2011). Incidence of various cardiac arrhythmias and conduction disturbances due to high dose intravenous methylprednisolone in patients with multiple sclerosis. J. Neurol. Sci..

[114] Osuagwu F., Jahnke B. (2016). Intravenous Methylprednisolone–Induced Nocturnal Sinus Bradycardia in a Multiple Sclerosis Patient. Prim. Care Companion CNS Disord..

[115] Taylor M.R., Gaco D. (2013). Symptomatic Sinus Bradycardia After a Treatment Course Of High-dose Oral Prednisone. J. Emerg. Med..

[116] Pavičić T., Ruška B., Adamec I., Habek M. (2019). Recurrent atrial fibrillation after pulse corticosteroid treatment for a relapse of multiple sclerosis. Mult. Scler. Relat. Disord..

[117] Frahm N., Hecker M., Zettl U.K. (2019). Polypharmacy in outpatients with relapsing-remitting multiple sclerosis: A single-center study. PLoS ONE.

[118] Piotrowicz E., Baranowski R., Piotrowska M., Zieliński T., Piotrowicz R. (2009). Variable Effects of Physical Training of Heart Rate Variability, Heart Rate Recovery, and Heart Rate Turbulence in Chronic Heart Failure. Pacing Clin. Electrophysiol..

[119] Ziemssen T., Siepmann T. (2019). The Investigation of the Cardiovascular and Sudomotor Autonomic Nervous System-A Review. Front. Neurol..

[120] Jagannath V.A., Pucci E., Asokan G.V., Robak E.W. (2019). Percutaneous transluminal angioplasty for treatment of chronic cerebrospinal venous insufficiency (CCSVI) in people with multiple sclerosis. Cochrane Database Syst. Rev..

