# Supplementary material for: Cardiac Autonomic Dysfunction in Multiple Sclerosis: A Systematic Review of Current Knowledge and Impact of Immunotherapies
Source: J Clin Med. 2020 Jan 24;9(2):335. doi: 10.3390/jcm9020335 (PMC7073977; doi:10.3390/jcm9020335)
Supplement: Supplementary file 1 [file jcm-09-00335-s001.pdf]

Supplement 1

Articles excluded after full text review

Review articles

Aguiar C, Batista S, Pacheco R. Cardiovascular effects of fingolimod: Relevance, detection and approach. Rev Port Cardiol. 2015 Apr;34(4):279-85.

Gajofatto A. Spotlight on siponimod and its potential in the treatment of secondary progressive multiple sclerosis: the evidence to date. Drug Des Devel Ther. 2017 Nov 2;11:3153-3157.

Haensch CA, Jörg J. Autonomic dysfunction in multiple sclerosis. J Neurol. 2006 Feb;253 Suppl 1:I3-9.

Kaplan TB, Berkowitz AL, Samuels MA. Cardiovascular Dysfunction in Multiple Sclerosis. Neurologist. 2015 Dec;20(6):108-14.

Miglis MG, Muppidi S. Autonomic dysfunction in multiple sclerosis and other updates on recent autonomic research. Clin Auton Res. 2018 Aug;28(4):391-393.

Sternberg Z. Genetic, Epigenetic, and Environmental Factors Influencing Neurovisceral Integration of Cardiovascular Modulation: Focus on Multiple Sclerosis. Neuromolecular Med. 2016 Mar;18(1):16-36.

Vargas WS, Perumal JS. Fingolimod and cardiac risk: latest findings and clinical implications. Ther Adv Drug Saf. 2013 Jun;4(3):119-24.

Ward MD, Jones DE, Goldman MD. Overview and safety of fingolimod hydrochloride use in patients with multiple sclerosis. Expert Opin Drug Saf. 2014 Jul;13(7):989-98.

Wens I, Eijnde BO, Hansen D. Muscular, cardiac, ventilatory and metabolic dysfunction in patients with multiple sclerosis: Implications for screening, clinical care and endurance and resistance exercise therapy, a scoping review. J Neurol Sci. 2016 Aug 15;367:107-21.

Editorials/Comments

Carroll WM. Oral therapy for multiple sclerosis--sea change or incremental step? N Engl J Med. 2010 Feb 4;362(5):456-8.

Mori M. Lethal arrhythmia due to fingolimod, a S1P receptor modulator: are we overestimating or underestimating? J Neurol Neurosurg Psychiatry. 2015 Aug;86(8):823.

Out of scope of review

Barry A, Cronin O, Ryan AM, et al. Cycle ergometer training enhances plasma interleukin-10 in multiple sclerosis. Neurol Sci. 2019 Sep;40(9):1933-1936

Christiansen CF, Christensen S, Farkas DK, et al. Risk of arterial cardiovascular diseases in patients with multiple sclerosis: a population-based cohort study. Neuroepidemiology. 2010;35(4):267-74.

Cornblath DR, Bienen EJ, Blight AR. The safety profile of dalfampridine extended release in multiple sclerosis clinical trials. Clin Ther. 2012 May;34(5):1056-69.

Dhar TG, Xiao HY, Xie J, et al. Identification and Preclinical Pharmacology of BMS-986104: A Differentiated S1P1 Receptor Modulator in Clinical Trials. ACS Med Chem Lett. 2016 Jan 19;7(3):283-8.

Egom EE, Kruzliak P, Rotrekl V, Lei M. The effect of the sphingosine-1-phosphate analogue FTY720 on atrioventricular nodal tissue. J Cell Mol Med. 2015 Jul;19(7):1729-34.

Jakimovski D, Gandhi S, Paunkoski I, et al. Hypertension and heart disease are associated with development of brain atrophy in multiple sclerosis: a 5-year longitudinal study. Eur J Neurol. 2019 Jan;26(1):87-e8.

Lott D, Lehr T, Dingemanse J, Krause A. Modeling Tolerance Development for the Effect on Heart Rate of the Selective S1P1 Receptor Modulator Ponesimod. Clin Pharmacol Ther. 2018 Jun;103(6):1083-1092.

Pinto AP, Guimarães CL, Souza GADS, et al. Sensory-motor and cardiorespiratory sensory rehabilitation associated with transcranial photobiomodulation in patients with central nervous system injury: Trial protocol for a single-center, randomized, double-blind, and controlled clinical trial. Medicine (Baltimore). 2019 Jun;98(25):e15851.

Pirttisalo AL, Sipilä JOT, Soilu-Hänninen M, et al. Adult hospital admissions associated with multiple sclerosis in Finland in 2004-2014. Ann Med. 2018 Jun;50(4):354-360.

Spiegelstein O, Mimrod D, Rabinovich L, et al. A Thorough QT/QTc Study With Laquinimod, a Novel Immunomodulator in Development for Multiple Sclerosis and Huntington Disease. Clin Pharmacol Drug Dev. 2019 Jan;8(1):49-59.

Sternberg Z. Impaired Neurovisceral Integration of Cardiovascular Modulation Contributes to Multiple Sclerosis Morbidities. Mol Neurobiol. 2017 Jan;54(1):362-374.

Vollmer T, Henney HR 3rd. Pharmacokinetics and tolerability of single escalating doses of fampridine sustained-release tablets in patients with multiple sclerosis: a Phase I-II, open-label trial. Clin Ther. 2009 Oct;31(10):2206-14.

No relevant information

Comi G, Patti F, Rocca MA, et al. Efficacy of fingolimod and interferon beta-1b on cognitive, MRI, and clinical outcomes in relapsing-remitting multiple sclerosis: an 18-month, open-label, rater-blinded, randomised, multicentre study (the GOLDEN study). J Neurol. 2017 Dec;264(12):2436-2449.
